# Knobloch syndrome associated with Polymicrogyria and early onset of retinal detachment: two case reports

**DOI:** 10.1186/s12886-017-0615-z

**Published:** 2017-11-25

**Authors:** Robert J. White, Yao Wang, Peter Tang, Sandra R. Montezuma

**Affiliations:** Department of Ophthalmology and Visual Neurosciences, University of Minnesota, 420 Delaware St. SE, MMC 493, Minneapolis, MN 55455-0501 USA

**Keywords:** Knobloch syndrome, *COL18A1*, Retinal detachment, Polymicrogyria, Case report

## Abstract

**Background:**

Knobloch Syndrome (KS) is a rare congenital syndrome characterized by occipital skull defects and vitreoretinal degeneration. Retinal detachment (RD) often occurs at the end of the first decade of life or later. Aside from occipital skull defects, central nervous system abnormalities are uncommon.

**Case presentations:**

We report on two siblings with KS. The first, a seven month old male, presented with nystagmus and was found to have a serous RD and a tessellated retinal appearance. His sister had a history of multiple visual abnormalities and had a similar retinal appearance although no signs of RD, but retina staphylomas. Genetic testing performed on both siblings showed a mutation in *COL18A1*, diagnostic of KS. MRI of both siblings demonstrated polymicrogyria but did not show occipital defects.

**Conclusions:**

Although several families with KS have been described previously, our case is noteworthy for several reasons. The RD observed in our first patient occurred at an early age, and we find evidence of only one patient with KS who had an RD identified at an earlier age. The findings of polymicrogyria are not characteristic of KS, and we found only a few previous reports of this association. Additionally, we review potential treatment options for this condition.

## Background

Knobloch Syndrome (KS) is a rare autosomal recessive syndrome first described in 1971 characterized by vitreoretinal degeneration and occipital skull abnormalities [1]. Clinical heterogeneity is present, although virtually all patients have ocular abnormalities that typically result in bilateral loss of vision. Ophthalmic findings include retinal detachment (RD), high myopia, early-onset cataracts, pigment dispersion, congenital glaucoma, and lens subluxation.

Midline occipital defects, namely bone defects, encephalocele, or aplasia cutis congenita, are characteristic findings. Other central nervous system findings are overall rare and not considered to be stereotypic features of KS. Caglayan et al. review seven cases of patients with KS associated with other central nervous system findings including pachygyria, polymicrogyria and cerebellar atrophy among other findings [2]. Developmental delay is observed in only a minority of patients, although is observed more frequently in patients who also possess central nervous system abnormalities [2, 3]. Other less common findings include seizures, hyperextensibility of joints, lung hypoplasia, cardiac dextroversion, midface hypoplasia, flat nasal bridge, and duplicated renal collecting system observed in single families [4].

The causative gene in KS has been identified as *COL18A1*, which encodes for collagen type XVIII α-1 chain. It is ubiquitously expressed in vascular and epithelial basement membranes and has multiple functions in ocular and neurologic development including maintenance of the basement membrane, cell proliferation, and angiogenesis [5].

Herein, we describe two siblings with KS associated with polymicrogyria, an anomaly sporadically associated with KS [2, 6]. Polymicrogyria is a condition characterized by multiple small gyri leading to an abnormally thick cerebral cortex. It presents with variety of clinical symptoms dependent on the specific region of the brain that is affected, although seizures and developmental delay are commonly described. Our first case is also noteworthy as the patient presented with a RD at only 7-months-old, which to our knowledge is the second youngest age reported to date in a patient with KS [7]. We then briefly describe potentially promising treatment options for KS.

## Case presentations

### Case 1

**Fig. 1:**
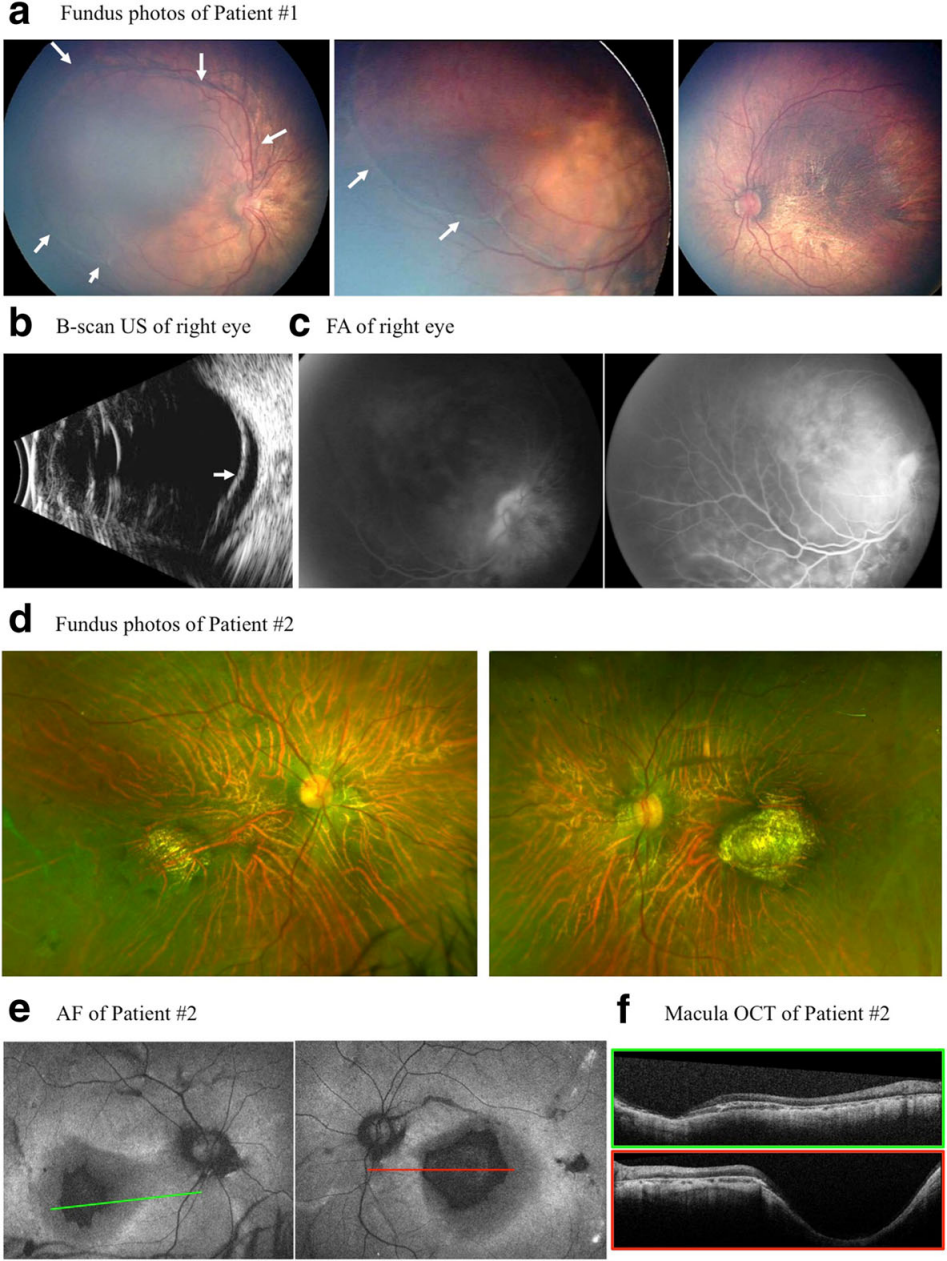
**a** Fundus photo of the right eye (OD) of Patient 1 shows a tilted optic nerve with trace pallor and a large posterior serous retinal detachment (RD) of the macula with surrounding demarcation lines and a subretinal fibrotic band. The remainder of the retina appears thin and atrophic. Left eye (OS) shows a tilted optic nerve with pigment mottling and central macular atrophy but no evidence of a serous RD. **b**. B-scan of Patient 1 shows subretinal fluid OD. **c** Fluorescein angiography (FA) of Patient 1 shows posterior pooling with early and late optic nerve hyperfluorescence OD. **d**. Fundus exam of Patient 2 shows mild optic disc pallor, retinal pigment epithelial atrophy, mild staphyloma, vascular attenuation, and a fundus tigroidal appereance of both eyes (OU). **e** Fundus autofluorescence (FAF) of Patient 2 shows significant macular RPE atrophic changes OU with significant hypoautofluorescence within the fovea and parafoveal region. **f** Optical coherence tomography of Patient 2 showing a mild staphyloma OD, moderate staphyloma OS, and irregular choriocapillaris with diffuse retinal thinning OU

A 7-month-old Hispanic male was referred for nystagmus. He was born at term and exhibited normal growth but had motor and social developmental delay. Family history was significant for an older sister (Case 2) with visual abnormalities. His parents and two older brothers did not have any clinically significant visual problems. On examination, the patient fixed and followed with the left eye (OS) but not with the right eye (OD). Cycloplegic refraction (CR) was found to be −2.50 + 3.50 × 090° OD and −7.00 + 3.50 × 090° OS. Anterior segment examination was unremarkable for both eyes (OU). Funduscopic examination OD showed a tilted optic nerve with trace pallor and a large posterior RD involving the macula with surrounding demarcation lines and a subretinal fibrotic band (Fig. 1a). The remainder of the retina appeared thin and atrophic. No retinal tears or holes were identified. Fundus examination OS showed a tilted optic nerve, a tessellated retinal appearance, retinal pigment epithelium (RPE) mottling, and central macular atrophy. There was no evidence of a retinal tear or detachment.

**Fig. 2:**
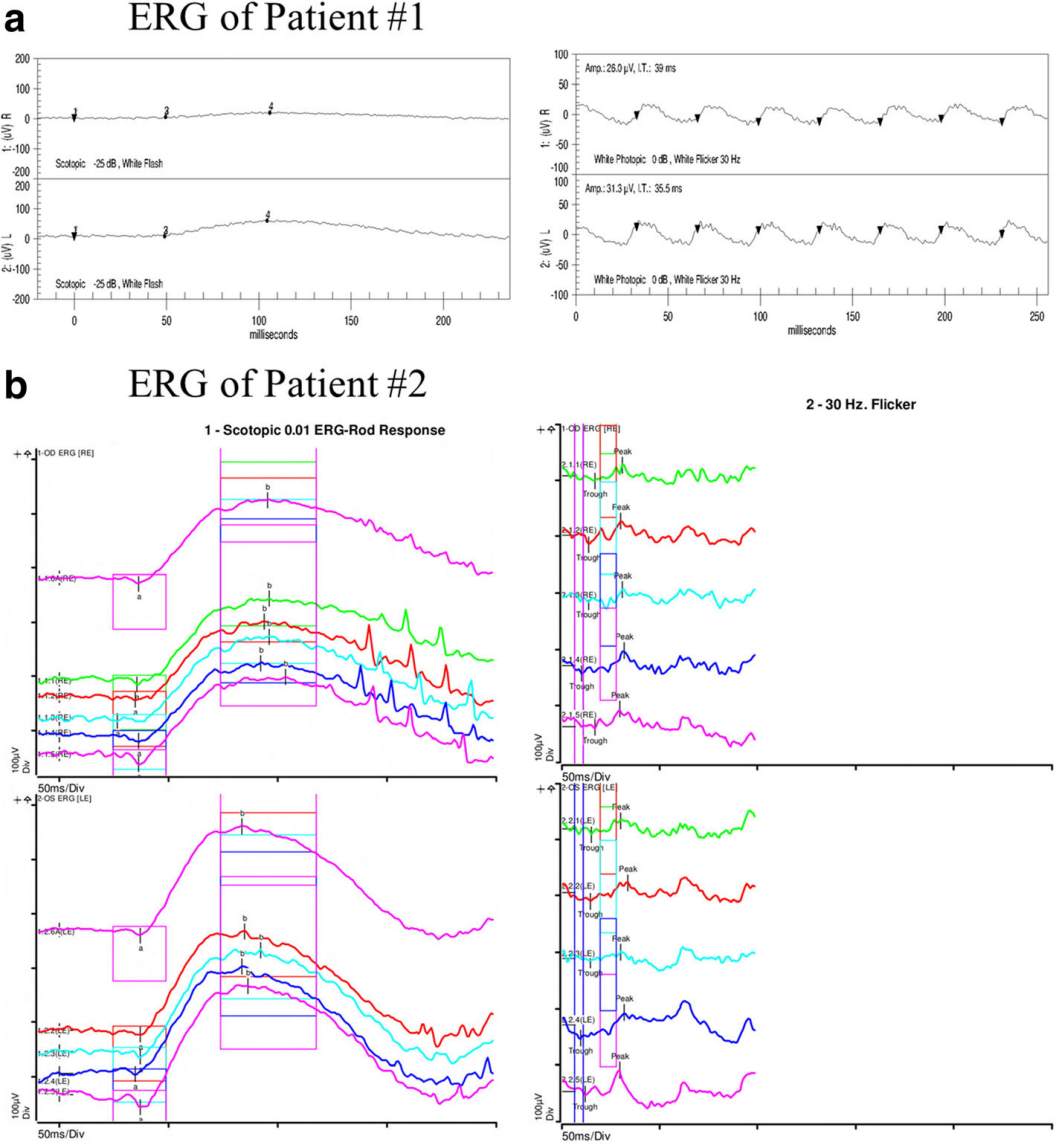
Full field electroretinograms (ERG), performed according to ISCEV standards. **a** ERG of Patient 1 performed under general anesthesia shows moderate to severely depressed responses from both cone and rod systems that are greater than could be attributed to anesthesia, myopic refractive error, partial retinal detachment, or mild supraduction. **b** Full field ERG of Patient 2 shows decreased amplitudes and delayed implicit times of the cone more than the rod system of both eyes. This ERG is consistent with cone-rod dystrophy

**Fig. 3:**
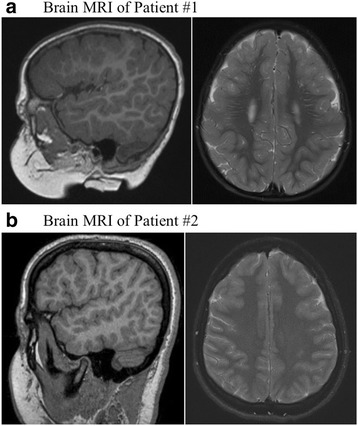
Brain magnetic resonance imaging findings. **a** Sagittal T_1_-weighted and axial T_2_-weighted images of patient 1 demonstrating gray matter thickening in the frontal gyri bilaterally with scattered areas of increased T_2_ signal intensity in the subcortical white matter consistent with polymicrogyria. There is no evidence of encephalocele. **b** Sagittal T_1_-weighted and axial T_2_-weighted images of patient 2 demonstrating gray matter thickening in the inferior and middle frontal gyri bilaterally consistent with polymicrogyria. There is no evidence of encephalocele

B-scan ultrasound OD confirmed a posterior RD (Fig. 1b). Fluorescein angiography (FA) OD demonstrated early and late hyperfluorescence consistent with pooling (Fig. 1c). FA OS only demonstrated RPE window defects and staining of drusen-like deposits. A full field electroretinogram (ERG) was performed according to ISCEV standards with the 2009 LKC machine and a small, infant Burian-Allen contact lens electrode under general anesthesia. This ERG showed moderately to severely depressed responses from both the cone and rod systems. The depressed responses were greater than the ones that could be attributed to anesthesia, myopic refractive error or partial retinal detachment (Fig. 2a). Optical coherence tomography (OCT) of OD showed an elevated retina with subretinal fluid; OS revealed RPE changes and thinning. A hereditary retinal dystrophy panel, covering roughly 180 genes, was significant for a mutation in the CNGB3 gene associated with achromatopsia. This, however, was inconsistent with the clinical presentation. Subsequent whole exome and mitochondrial DNA sequencing demonstrated a homozygous mutation in the *COL18A1* gene (NM_130445.3:c.2970_2971delAGinsC) associated with KS. Magnetic resonance imaging (MRI) of the brain demonstrated findings consistent with polymicrogyria but no evidence of an encephalocele (Fig. 3a). Fundus examination of the parents was unremarkable.

### Case 2

This 13-year-old female was the sibling of the 7-month-old boy from Case 1. She had a past ocular history significant for an ERG demonstrating mild cone-rod dystrophy, bilateral macular chorioretinal staphylomas, subnormal visual acuity, high myopia, and strabismus status post extraocular muscle surgery OU. Her medical history was significant for a history of precocious puberty at age 9. Her vision had been poor since birth but remained stable at 20/200 OU for the past 8 years. She was being reevaluated in light of her brother’s presentation. CR was found to be −9.00 + 0.50 × 110° OD and −11.50 + 2.00 × 080° OS with best-corrected visual acuity of 20/200 OU. On anterior segment exam there were bilateral patchy cortical cataracts. Fundus exam OU showed mild optic disc pallor, macular RPE atrophic changes, macular staphyloma, vascular attenuation, and a fundus with a tessellated appearance (Fig. 1d). Fundus autofluorescence confirmed significant RPE atrophic changes within the foveal and parafoveal regions of both eyes (Fig. 1e). OCT showed a mild staphyloma OD, moderate staphyloma OS, and irregular choriocapillaris with diffuse retinal thinning OU (Fig. 1f). A full field ERG was performed according to ISCEV standards using ESPION E3 system and DTL fiber recording electrodes. The ERG showed decreased amplitudes and delayed implicit times of the cone more than the rod system in both eyes. This ERG was consistent with cone-rod dystrophy that was stable compared to ERG obtained 4 years prior (Fig. 2b). Similar to her sibling from Case 1, the patient was found to have an identical homozygous mutation in the *COL18A1* gene, and subsequent brain MRI showed findings consistent with polymicrogyria without evidence of an encephalocele (Fig. 3b).

## Discussion and conclusions

In this paper, we report two siblings who presented with poor BCVA along with high myopia and anisometropia. Retinal examination and OCT demonstrated thinning of the RPE and an atrophic appearance, with a serous RD observed in one child although no leakage was seen on FA. ERG in both patients demonstrated significant depression of the cone and rod system. Whole genome and mitochondrial DNA sequencing eventually uncovered a mutation in homozygous mutation in the *COL18A1* gene, diagnostic of KS. Notably, neither patient had the characteristic encephalocele and both had polymicrogyria demonstrated on MRI.

Our findings add to the literature supporting the spectrum of brain anomalies observed with KS, including polymicrogyria. Additionally, our cases are consistent with other reported cases of KS with polymicrogyria in which polymicrogyria did not occur with midline occipital defects [2, 6]. Therefore, head imaging may be helpful in the diagnosis of KS and associated CNS abnormalities in patients with characteristic retinal findings but lacking an encephalocele. While the patient in Case 1 did experience delay in motor and social development, the patient in Case 2 experienced normal developmental milestones. To our knowledge, neither patient has any other neurologic abnormalities. In previously reported cases of KS with associated polymicrogyria, developmental delay was observed in most patients [2, 6].

The onset of RD at seven months of age in Case 1 was earlier than what is typically reported, as RDs tend to occur at the end of the first decade of life or later in patients with KS. There was one reported case of RD in the setting of KS identified at one month of age [7] and another case identified “before the age of one” [8]. KS is typically associated with rhegmatogenous RD, consistent with the associated vitreoretinal degeneration, as opposed to the serous RD observed in our patient [9]. There is at least one prior case describing a serous RD occurring in a child with KS [10]. Unfortunately however, the majority of case reports on patients with early onset of RD do not comment on the subtype of RD. [6–8, 11] The finding of a serous retinal detachment is of interest, as vitreoretinal degeneration would typically result in a rhegmatogenous RD. We find no basic science research to suggest a potential pathogenesis of serous RD development in patients with KS.

In the absence of obvious neurologic symptoms, the differential diagnosis of KS includes but is not limited to cone-rod dystrophy, Leber congenital amaurosis, retinitis pigmentosa, microcephaly lymphedema chorioretinal dysplasia syndrome, and Stickler syndrome. Khan et al. suggest that a triad of smooth iridies, ectopia lentis, and characteristic vitreoretinal degeneration is pathognomonic of KS based on an observation of eight children [10]. Notably, these findings were demonstrated in patients with an already known diagnosis of KS. We argue that the clinical triad described by Khan et al. is challenging to utilize within the clinical setting with an unknown diagnosis, and genetic testing is often essential for diagnosis. However, once a molecular diagnosis is reached, the patient should be reassessed to address possible associated ocular conditions of KS including pigment dispersion syndrome, RD, lens subluxation and cataracts [6]. In addition, it is important to emphasize that the genetic testing results need to be correctly interpreted and correlate with the clinical findings to avoid misleading diagnosis, as in our first patient his initial retinopathy panel revealed a mutation in the CNGB3 gene associated with achromatopsia. The lack of correlation of this condition with his clinical findings led to additional genetic testing with subsequent whole exome and mitochondrial DNA sequencing demonstrating a mutation in the *COL18A1*.

Although we contemplated repairing the serous RD in our patient, the prognosis for KS patients is often poor as could lead to the need for multiple interventions. Moysidis et al. describes a child with KS who underwent repair of a RD at 24 months of age and was also prophylactically treated with scleral buckle implantation [11]. Four years later, the patient is still doing well without evidence of recurrent RD, suggesting that this represents a potentially promising surgical prophylactic option. Given that these patients have high risk of RD during their life time, we offered to the parents treatment options of peripheral laser retinopexy with and without scleral buckle surgery vs cryopexy to the periphery. In Case 1, the parents elected for observation. They did however agree to have peripheral cryo-retinopexy OD only to prevent possible progression of the RD.

While treatment for KS is often supportive, recent advancements in our understanding of the pathophysiology of the disease come from studies in *Drosophila* [12]. Mutation of the *COL18A1* gene resulted in mitochondrial structural disorganization that caused a decrease in energy generation and enhanced reactive oxygen species (ROS) production. Interestingly, treating the mutants with the angiotensin II type 1 receptor antagonist losartan, a conventional hypertensive medication, has been shown to attenuate mitochondrial ROS production, improve mitochondrial morphology and restore function, suggesting a viable avenue for further investigation. Considerable research interest in the ocular renin-angiotensin system and its role in disease may help guide future treatment options for patients with KS [13].

Further investigation is necessary to enhance our understanding of the pathophysiology of KS so that we may offer improved medical and surgical treatments for our patients.

## Abbreviations

CR: cycloplegic refraction
ERG: electroretinogram
KS: Knobloch Syndrome
MRI: magnetic resonance imaging
OCT: optical coherence tomography
OD: right eye
OS: left eye
OU: both eyes
RD: retinal detachment
ROS: reactive oxygen species
RPE: retinal pigment epithelium
